# Perinatal Cells: A Promising COVID-19 Therapy?

**DOI:** 10.3389/fbioe.2020.619980

**Published:** 2021-01-14

**Authors:** Andrea Papait, Anna Cargnoni, Michal Sheleg, Antonietta R. Silini, Gilad Kunis, Racheli Ofir, Ornella Parolini

**Affiliations:** ^1^Department of Life Science and Public Health, Università Cattolica del Sacro Cuore, Rome, Italy; ^2^Centro di Ricerca E. Menni, Fondazione Poliambulanza-Istituto Ospedaliero, Brescia, Italy; ^3^Pluristem Ltd., Haifa, Israel; ^4^Fondazione Policlinico Universitario “Agostino Gemelli” IRCCS, Rome, Italy

**Keywords:** coronavirus-induced disease 2019, severe acute respiratory distress syndrome coronavirus-2, mesenchymal stromal cells, PLacental eXpanded, perinatal

## Abstract

The COVID-19 pandemic has become a priority in the health systems of all nations worldwide. In fact, there are currently no specific drugs or preventive treatments such as vaccines. The numerous therapies available today aim to counteract the symptoms caused by the viral infection that in some subjects can evolve causing acute respiratory distress syndromes (ARDS) with consequent admission to intensive care unit. The exacerbated response of the immune system, through cytokine storm, causes extensive damage to the lung tissue, with the formation of edema, fibrotic tissues and susceptibility to opportunistic infections. The inflammatory picture is also aggravated by disseminated intravascular coagulation which worsens the damage not only to the respiratory system, but also to other organs. In this context, perinatal cells represent a valid strategy thanks to their strong immunomodulatory potential, their safety profile, the ability to reduce fibrosis and stimulate reparative processes. Furthermore, perinatal cells exert antibacterial and antiviral actions. This review therefore provides an overview of the characteristics of perinatal cells with a particular focus on the beneficial effects that they could have in patients with COVID-19, and more specifically for their potential use in the treatment of ARDS and sepsis.

## SARS-CoV2 Pathology and the Rationale for the Use of MSCs in the Treatment of COVID-19 Patients

The Severe Acute Respiratory Syndrome CoronaVirus 2 (SARS-CoV-2) pandemic began in Wuhan, China, in December 2019, and rapidly spread worldwide. The disease caused by SARS-CoV-2 is called coronavirus disease 2019 (COVID-19) and by December 2020, the disease has reached more than 61 million cases, with over 1.4 million deaths and the numbers are constantly rising (https://COVID-19.who.int/).

Patients affected by COVID-19 infection report symptoms such as headache, fever, weakness, cough, general malaise, and shortness of breath (Chen et al., 2020). Although in many cases the disease does not lead to the development of symptoms, in around 5% of patients, especially the elderly, in the presence of co-morbidities like hypertension and diabetes, the disease can become critical, giving rise to acute respiratory distress syndrome (ARDS), pneumonia and multiple organ dysfunction requiring intensive care unit treatment (ICU) (Chen et al., 2020; Li et al., 2020). The mortality rate ranges between 1 and 4% (Guan et al., 2020; Rothan and Byrareddy, 2020), with the highest percentage in the aged and most critical patients >80 years old (Chen et al., 2020).

Many of the observed symptoms in ICU patients are attributable to a widespread and uncontrolled inflammatory response that gives rise to cytokine storm, as well as a disseminated intravascular coagulopathy (DIC) and multiorgan failure (Li et al., 2020).

As a matter of fact, screenings conducted on ICU patients showed that severe COVID-19 patients had high serum levels of inflammatory cytokines, such as tumor necrosis factor-alpha (TNF-α), interleukin-1 beta (IL-1β), IL-6, IL-17, interferon-gamma (IFNγ), granulocyte colony-stimulating factors (GM-CSF) and chemokine like the macrophage inflammatory proteins 1-α (MIP-1 α) (De Biasi et al., 2020; Huang et al., 2020; Liu J. et al., 2020).

Moreover, many patients also tend to develop a symptomatic framework that meets the diagnostic criteria for sepsis and septic shock according to the Sepsis-3 International Consensus (Singer et al., 2016). Importantly, following the inflammatory phase due to the dysregulated immune response, many patients develop lymphopenia, and thus are subject to other infections due to lowered immune response (Tan et al., 2020).

At the moment there is no specific treatment for SARS-CoV-2 infection and its consequences. Several clinical trials have been conducted using drugs targeting cytokines responsible for the observed adverse effects such as tocilizumab that targets IL-6 (Guaraldi et al., 2020; Stone et al., 2020), or anakinra to counteract the effects related to IL-1β (NCT04443881, NCT04603742, Huet et al., 2020). Other antiviral drugs such as Remdesivir are still present in therapeutic protocols today, finding an application in limiting viral replication (Beigel et al., 2020).

Mesenchymal stromal cells (MSCs) represent a possible alternative treatment for COVID-19 aimed at counteracting the damage and organ failure due to inflammatory and fibrotic processes induced by SARS-CoV2, as well as responding to many of the symptoms induced by the viral infection. In fact, MSCs have been studied for years in regenerative medicine for the immunomodulatory properties that make them suitable for the treatment of many chronic or degenerative diseases, where the immune response is dysregulated (Shi et al., 2018; Koliaraki et al., 2020). Furthermore, these cells are also characterized by antimicrobial properties (Alcayaga-Miranda et al., 2017) and reduced expression of HLA-II strongly reducing the risk of allograft rejection (Ryan et al., 2005).

Initially isolated from the bone marrow over the years they have been isolated from different tissues such as adipose tissue, dental pulp, and perinatal tissues (Silini et al., 2020). In particular, in this review we will focus our attention on the potential application of perinatal derived cells (PDCs) in the treatment of COVID-19 with a focus on their anti-inflammatory and antifibrotic properties, aimed at counteracting ARDS, and on their antimicrobic and antiviral properties.

## Perinatal Tissues as a Precious Cell Source

The use of cell therapy has attracted much attention in the scenario of regenerative medicine due to its potential therapeutic benefits. Indeed, living cells provide multifaceted mechanisms of action that can tackle a broad spectrum of deregulated physiological processes especially in degenerative and inflammatory diseases.

One of the most intriguing sources for cell therapy that has attracted much attention in the past decade is perinatal tissues. The term “perinatal” refers to birth-associated tissues that are obtained from term placentas (including chorionic villi, chorionic plate) and fetal annexes (that include amniotic and chorionic membrane, amniotic fluid, and umbilical cord). Indeed, perinatal tissues have proven to be precious reservoirs of cells (In 't Anker et al., 2004; Wang et al., 2004; De Coppi et al., 2007; Parolini et al., 2008; Abumaree et al., 2013).

Initially, perinatal tissues drew attention as an interesting cell source due to their early embryological origin suggesting that perinatal cells could possess unique plasticity and differentiation properties (Parolini and Soncini, 2006). In addition, when compared to other cell sources such as bone marrow, practical and logistical reasons made the placenta an attractive cell source. It is easily obtained after birth without invasive procedures, and it is considered biological waste thus bypassing ethical tensions associated to other cell sources.

Nowadays, perinatal-derived cells (PDCs) have obtained significant recognition for their promising applications in regenerative medicine (Silini et al., 2015). As a matter of fact, during this past decade the literature published on PDCs has grown exponentially, substantiating the interest in using cells from these tissues and, most importantly, demonstrating that indeed PDCs are important contenders with stromal cells from other tissues, such as bone marrow- or adipose-derived mesenchymal stromal cells (MSCs). Below we will briefly discuss why perinatal cells represent valid alternatives.

First, there is strong evidence that PDCs from both maternal and fetal tissues do not induce an immune response *in vitro* (Bailo et al., 2004; Wolbank et al., 2007; Banas et al., 2008; Magatti et al., 2008; Weiss et al., 2008; Prasanna et al., 2010; Tipnis et al., 2010; Papait et al., 2020), making them attractive for allogeneic transplantation. Second, in order to become fully immunosuppressive, several studies indicate that MSCs from bone marrow require “licensing” with inflammatory stimuli such as IFNγ and TNFα (Krampera et al., 2006; Ren et al., 2008; Sheng et al., 2008; Mougiakakos et al., 2011; Shi et al., 2012). Accordingly, bone marrow MSCs cultured in transwell, and the secretome from bone marrow MSCs, are not able to exert suppressive effects if they are not previously exposed to inflammatory stimuli (Krampera et al., 2003, 2006; Groh et al., 2005). In the case of PDCs, “licensing” does not seem to be mandatory for their suppressive effects (Magatti et al., 2008; Rossi et al., 2012; Lange-Consiglio et al., 2020; Papait et al., 2020) although stimulation of PDCs with pro-inflammatory cytokines has been shown to increase secretome potency (Allen et al., 2018). In line with this, the secretome from unstimulated PDCs exerts significant immunomodulatory effects *in vitro* (Rossi et al., 2012; Pianta et al., 2015; Magatti et al., 2016; Papait et al., 2020) and therapeutic effects *in vivo* in diseases with a deregulated inflammatory response (Cargnoni et al., 2012, 2014; Roy et al., 2013; Danieli et al., 2015; Chatterjee et al., 2016; Magatti et al., 2016; Van Linthout et al., 2017; Giampa et al., 2019), altogether suggesting that these cells constitutively secrete bioactive factors that promote regeneration.

Third, PDCs and their secretome have robust therapeutic properties when transplanted in animal models of different pathological conditions, such as inflammatory disorders, autoimmune diseases, neurodegenerative diseases, ischemia/reperfusion injuries, diabetes, and liver and lung fibrosis. The controversial ability of PDCs to differentiate *in vivo*, as well as the low survival and engraftment in host tissues after transplantation, suggest that they do not act via stemness properties to replace injured tissue but rather act via paracrine mechanisms on tissue resident cells or on immune cells located in the injured tissue. PDCs can create a reparative environment through cell-to-cell contact and the secretion of growth factors, cytokines, and the release of extracellular vesicles (containing peptides/proteins, mRNA, and microRNA). These in turn can suppress the activation/function of inflammatory cells; prevent apoptosis and promote the survival of damaged tissue cells, reduce oxidative injury often involved in tissue damage, and favor angiogenesis.

The immunomodulatory activity of PDCs includes inhibition of immune cell (e.g., T and B lymphocyte) proliferation (Magatti et al., 2008, 2020; Weiss et al., 2008; Najar et al., 2010a; Prasanna et al., 2010; Raicevic et al., 2011; Zhou et al., 2011; Roy et al., 2013; Li et al., 2014; Wang et al., 2014; Pianta et al., 2016; Cargnoni et al., 2020; Papait et al., 2020), inhibition of the maturation toward effector immune cells (cells which actively respond to an immunogenic stimuli) (Magatti et al., 2009; Pianta et al., 2016), expansion of regulatory immune cells that can suppress the immune response (Chang et al., 2006; Najar et al., 2010b; Parolini et al., 2014; Amari et al., 2015; Pianta et al., 2015, 2016; Papait et al., 2020), inhibition of the secretion of pro-inflammatory cytokines and chemokines (IL-1β, IL-2, TNF -α, and IFN-γ) (Kronsteiner et al., 2011; Kang et al., 2012; Karlsson et al., 2012; Parolini et al., 2014; Pianta et al., 2015), involved in the aggravation of the inflammatory response, and promotion of the production of anti-inflammatory factors (IL-10, TGF-β), (Pianta et al., 2015; Papait et al., 2020).

The promising findings obtained from preclinical studies, along with the high availability, excellent safety profile, lack of ethical challenges, and low immunogenicity, provide a strong rationale for investigating perinatal cells as a potential innovative therapy in humans.

## PDCs Applied in Clinical Trials for Pulmonary Disorders

Over the past decade a large number of clinical trials have emerged worldwide using cell-based therapies to treat various diseases (clinicaltrials.gov), including neurological disorders, muscle repair, cardiovascular diseases, immune related diseases, and lung diseases. Among these studies the use of PDCs is rapidly rising with only 1% of the studies using perinatal tissues as a source of cells, between the years 1994 and 2009, to almost 30% in 2018 (Verter et al., 2018; Moll et al., 2019).

**Figure 1:**
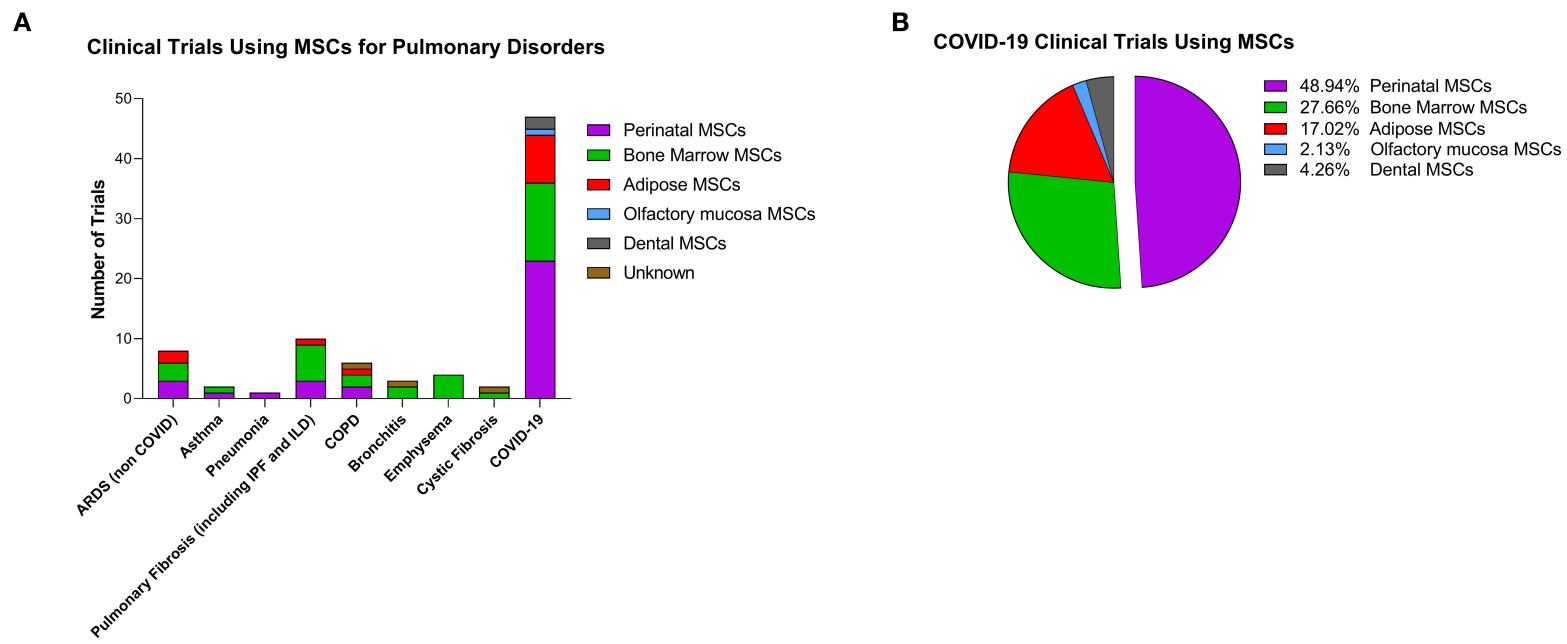
Clinical trials using cell-based therapy for pulmonary disorders. **(A)** Clinical trials as reported on clinicaltrials.gov (as of July 1, 2020) with stromal cells for pulmonary conditions. Colors represent the different cells used in the studies. ARDS, acute respiratory distress syndrome; IPF, idiopathic pulmonary fibrosis; ILD, interstitial lung disease; COPD, chronic obstructive pulmonary disease. **(B)** Percentage of MSCs from different tissues used in COVID-19 clinical trials.

According to a large analysis of 914 MSC trials reported through 2018 (Kabat et al., 2020), the three most common conditions for MSC trials are neurological, joint, and cardiovascular diseases which account for 42% of all trials. Pulmonary conditions account for only about 6% of all trials (Marquez-Curtis et al., 2015; Kabat et al., 2020). However, since the emergence of the novel coronavirus disease (COVID-19), that features pathological changes that are similar to acute lung injury with serious pulmonary inflammation and inflammatory cytokine storm, numerous clinical trials with MSCs as a new treatment for COVID-19 were registered (Liu S. et al., 2020). Indeed, as of July 2020 there were 83 clinical trials (clinicaltrials.gov) using MSCs for various immune/inflammatory pulmonary conditions (Figure 1A) and 57% of these (47 out of 83) were specifically for COVID-19. With respect to the source of the MSCs, perinatal tissues account for 40% of all pulmonary studies and 49% of the COVID-19 trials (Figure 1B). Results obtained from clinical trials which transplanted PDCs intravenously (IV) in patients with lung fibrosis have shown that these cells are safe with null/mild side effects. Specifically, in a phase 1 clinical trial, PDCs (PDA-001) infused in four patients with refractory pulmonary sarcoidosis produced a transient and mild increase in mean pulmonary artery pressure. In the year following treatment two of the patients showed improvement in their chest x-ray and had prednisone withdrawn (Baughman et al., 2015). Another phase 1b study using placental stromal cells to treat eight patients with mild to moderate idiopathic pulmonary fibrosis (IPF) demonstrated that 6 months after placental MSC infusion, lung function and CT fibrosis scores were stable from baseline, proving feasibility and short-term safety of placental MSCs (Chambers et al., 2014). Patients tolerated cell administration with only a transient fall of oxygen saturation but no changes in hemodynamics showing satisfactory short-term safety profile in IPF patients (Chambers et al., 2014).

In addition, a case report at the Siping Hospital of China Medical University described that, at the end of 12 month follow-up, a 56-year-old patient with IPF that was IV-injected with human umbilical cord MSCs showed a relevant reduction of oxygen therapy requirement, an improvement of physical performance and respiratory parameters (lung function and CT scores). No serious or clinically relevant side effects were observed during the entire study period (Zhang et al., 2017). Finally, a completed phase 1 clinical trial (NCT02277145), without published results, investigated the effects of clinical grade umbilical cord MSCs, injected directly into lung lesion by fiberoptic bronchoscopy in patients with radiation-induced pulmonary fibrosis. Six months from injection, the safety and effectiveness of the treatment will be evaluated through CT density histogram and the patients' self-evaluation.

Numerous clinical trials are currently evaluating the efficacy and safety of PDCs applied as treatment for acute respiratory distress syndrome (ARDS) induced by COVID-19 infection. Many countries promoted these studies including China, US, Europe (France, Spain, UK, Ukraine), and South America (Colombia). These studies, registered at www.clinicaltrials.gov, combine standard of care with the infusion of human umbilical cord or Wharton's jelly derived MSCs (hUC-MSCs and hWJ-MSCs, respectively), or with the intramuscular (IM) injection of placental expanded adherent stromal cells (NCT04614025, NCT04389450) to evaluate the ability of cell therapies to potentiate conventional treatment efficacy for COVID19. Out of these studies one study will use placenta-derived multipotent mesenchymal stromal cells (NCT04461925) and another, the REALIST study, will apply a selected cell population and specifically CD362 enriched hUC-MSCs to treat patients with moderate to severe ARDS (NCT03042143).

Most of these studies plan to implement multiple cell infusions with cell doses ranging from 1 × 10^6^ cells/kg (NCT04313322, NCT04429763, NCT03608592, NCT04339660, NCT04457609, NCT04461925, NCT04390139, NCT04333368), to 5 × 10^6^ cells/kg (NCT04273646) or independent from body weight, doses ranging from 4 to 5 × 10^7^ cells (NCT04288102, NCT04390152) and up to 1–6 × 10^8^ cells (NCT04456361, NCT04355728, NCT04389450). One of these studies is a Phase 1/2a dose-escalating study (NCT04452097) assessing the safety and the maximum tolerated dose of IV infused hUC-MSCs. In the phase 1 three doses will be infused 0.5 × 10^6^, 1.0 × 10^6^, or 1.5 × 10^6^ cells/kg/body weight with three patients/group; at day 3 the incidence of infusion-related adverse events will be evaluated and at day 28 the incidence of any treatment-emergent serious adverse events. In the phase 2a, a total of 30 subjects will be randomized into the treatment and control groups.

Almost all studies are aimed to assess the efficacy of cell therapy by evaluating intergroup (cell treated group vs. placebo group) differences for clinical parameters such as mortality rate, length of hospitalization, oxygenation index (PaO_2_/FiO_2_); one of these studies (NCT04339660) has the primary endpoint to assess the improvement of inflammatory parameters/factors such as plasma levels of TNF-α, IL-1β, IL-6, TGF-β, IL-8 and C-reactive protein (CRP).

Although most are Phase I or Phase I/II clinical trials, few of these (NCT04269525, NCT03042143, NCT04288102, NCT04429763, NCT04389450, NCT04614025) are Phase II trials that will collect preliminary data on cell therapy efficacy, in addition to data about safety and short-term adverse events.

In general, however, all these studies will involve a small numbers of patients, from a few participants (five to nine patients) to a maximum of 140 (NCT04389450), to have conclusive indications on PDCs' safety and efficacy in COVID-19 patients, cells will be administered in large, randomized, controlled, double-blinded trials with long-term follow-up.

Considering these important limitations, it cannot be ignored that some published reports of early phase studies performed in China show promising results from PDC therapy in severe cases of COVID-19. In a Phase I study (Meng et al., 2020), 18 patients with moderate-severe COVID-19 were enrolled and received standard COVID-treatment regimens, nine of them (the treated group), also received three IV infusions of hUC-MSCs (1 × 10^6^cells/each infusion) on days 0, 3, and 6. Two patients from the treated group suffered from transient facial flushing and fever, and another from transient hypoxia at 12 h post infusion. A reduced number of patients in treated group with respect to control group (1 vs. 4), required mechanical ventilation and experienced dyspnea (1 treated vs. 5 controls). Furthermore, hUC-MSC-treated patients showed a trend in reduction of serum IL-6. On the basis of these outcomes, the authors started a Phase 2 randomized placebo-controlled trial which will recruit 90 patients with severe COVID-19 (see NCT04288102).

From January to April 2020, another clinical study enrolled 31 patients with severe COVID-19, all treated with one to three doses of hUC-MSCs (1 × 10^6^cells/kg each dose) suspended in 100 ml saline (Guo et al., 2020). The authors observed no adverse events attributable to IV cell infusion. Four patients died, 27 were discharged with amelioration of clinical and hematologic parameters, but lack of the control group did not allow conclusions to be drawn on potential treatment efficacy.

Another study, published by Shu et al. (2020), enrolled 41 patients with severe COVID-19 who did not respond to a 7- to 10-day standard treatment. All patients received conventional treatment (ventilation, antiviral agents, antibiotic agents if needed, glucocorticoid therapy) and, in addition, a group of these patients (*n* = 12), received one IV infusion of hUC-MSCs (2 × 10^6^cells/kg in 100 ml saline). During 2 week-period of observation, patients who received hUC-MSCs, had no adverse reactions. In comparison with control group (*n* = 29), treated patients showed a shorter time to clinical improvement (9.0 vs. 14.0 days, *p* = 0.006), a higher percentage with significant remission of dyspnea and absorption of imaging (91.67 vs. 51.72%), a better oxygenation index, and a significant amelioration of CT scores, ground-glass opacity and consolidation, paralleled with reduced plasmatic levels of CRP and IL-6; all are parameters that indicate reduced lung inflammation. The authors speculate that hUC-MSCs can reduce lung inflammation by reducing the release of inflammatory cytokines through an immunomodulatory action.

In most of the completed and ongoing studies, COVID-19 patients received/will receive PDCs through IV infusion, however it is not yet clear whether the IV route is the best choice. COVID-19 can lead to a form of disseminated intravascular coagulation (DIC) and many of the critically ill COVID-19 patients with poor prognosis are in a systemic procoagulant state (Arachchillage and Laffan, 2020; Connors and Levy, 2020; Klok et al., 2020; Magro et al., 2020; Oxley et al., 2020; Spyropoulos et al., 2020; Tang et al., 2020; Wang T. et al., 2020; Zhang et al., 2020; Zhou et al., 2020), and MSC products are known to express variable levels of a highly procoagulant tissue factor (TF/CD142) (Morrissey, 2004). Therefore, alternative routes of cell administration such as the IM injection are increasingly explored. The IM route holds great advantages over other administration routes, such as the possibility to administer a higher number of cells and thus potentially increase efficacy (Braid et al., 2018; Caplan et al., 2019), and the vascularized muscle support provides a channel for systemic release of paracrine mediators. In addition, the large muscle tissue allows for multiple injection sites (Caplan et al., 2019; Hamidian Jahromi and Davies, 2019). IM delivery has been shown to be safe (reviewed in Caplan et al., 2019; Hamidian Jahromi and Davies, 2019) in several clinical studies (Winkler et al., 2018; Norgren et al., 2019) with placenta-derived mesenchymal-like cells [PLacental eXpanded (PLX)-PAD] and is now being tested in a double-blind, multicenter study to evaluate the efficacy of PLX-PAD for the treatment of COVID-19 (NCT04389450). PLX-PAD cells are adherent stromal cells isolated from the placenta of healthy women following a cesarean section. While PLX-PAD cells express membrane markers typical of classical MSCs, they have a minimal ability to differentiate *in vitro* into cells of the mesodermal lineage and are thus termed MSC-like cells. In addition to the previously mentioned phase II study, PLX-PAD are also being used (as of July 2020) to treat 20 COVID-19 patients in Israel under emergency/compassionate use, and in the USA under Single Patient IND for Compassionate or Emergency Use. Patients received either one or two treatments of 300 million cells administered via IM injections. Data published on the first eight patients describe an overall good safety profile and clinical improvement in several parameters such as CRP, PEEP, and P/F following PLX-PAD treatment. Specifically, levels of CRP in all patients were elevated prior to PLX-PAD treatment with levels ranged from 82 to 394 mg/L at time of treatment. Starting as early as 24 h post treatment, CRP levels decreased and have decreased by 45 and 77% by day 3 and by day 5, respectively (Barkama et al., 2020).

## PDCs in Lung Fibrosis

### Pulmonary Fibrosis and SARS-CoV-2 Infection

**Figure 2:**
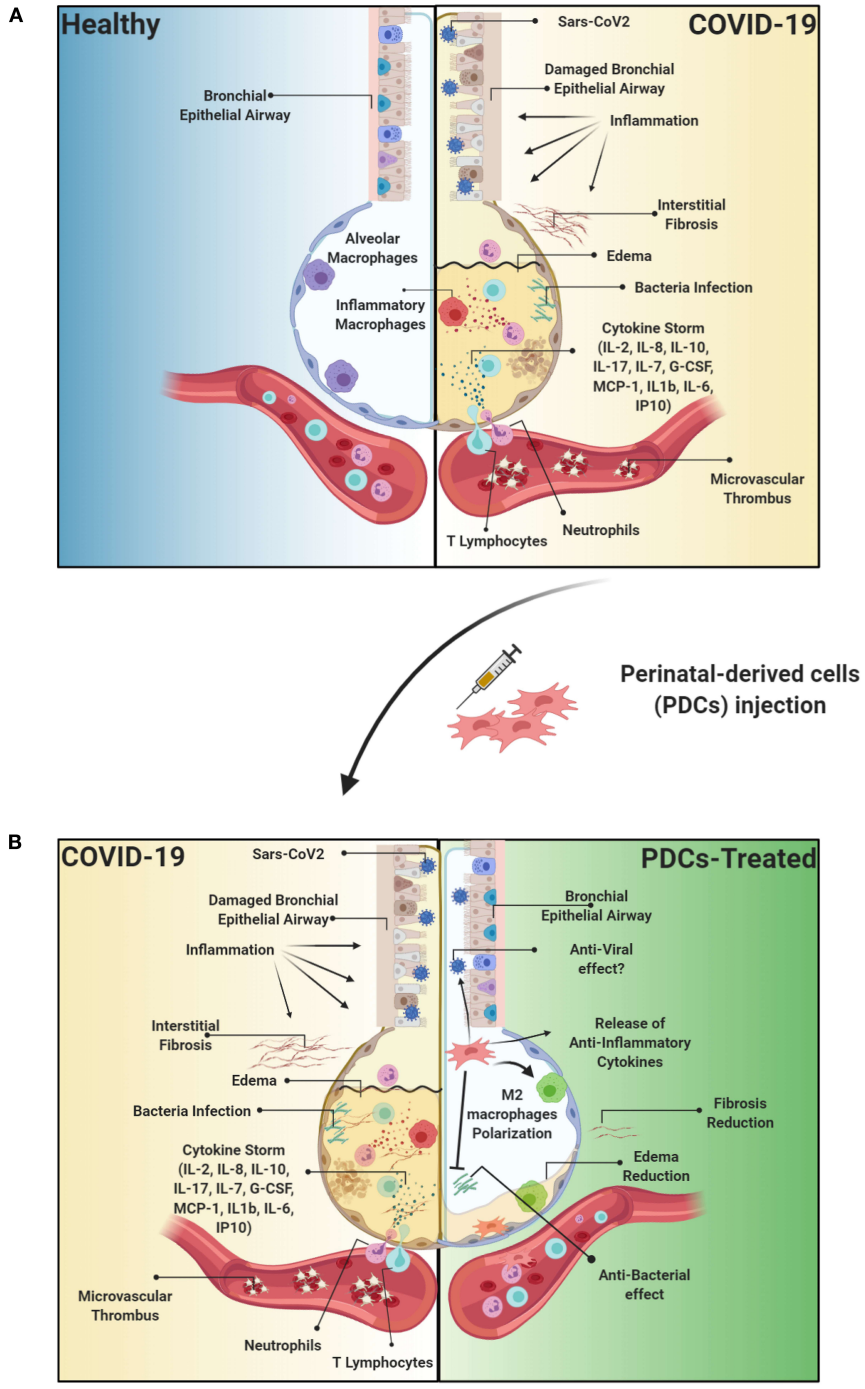
Etiopathology of the Sars-CoV2 viral infection and therapeutic effect of PDCs injection. **(A)** Sars-CoV2 is responsible for acute respiratory distress syndrome (ARDS) resulting in thousands of patients requiring intensive care unit (ICU) treatment. Importantly, the virus responsible of trigger an exacerbated immune response, resulting in a cytokine storm, and consequently to the damage of the epithelial airway, followed by lung edema, dysfunction of air exchange and the formation of interstitial fibrosis, thus creating an environment conducive to opportunistic bacterial infections or superinfection. Furthermore, COVID-19 is characterized also by the formation of vascular microthrombi that are often identified in areas of diffuse alveolar damage and are associated with diffuse endothelial damage. **(B)** PDCs treatment can directly affect the immune response through the release of immunomodulatory cytokines, such as IL-10, or anti-inflammatory molecules such as Prostaglandin (PG) E2, thus immunomodulating the exacerbated inflammatory response. Furthermore, MSCs can exert beneficial properties through the release of anti-microbial peptides such as LL-37. Moreover, PDCs can degrade or inhibit ARDS-induced fibrotic tissue by remodeling the extracellular matrix through the release of metalloprotease and TIMP, and modulation of the immune response can consequently reduce the formation of microthrombi affecting the alveolar capillaries. Created with Biorender.com.

Recent evidence has suggested a link between lung fibrosis and the SARS-CoV-2 virus infection. Indeed, COVID-19 patients experience a large spectrum of pulmonary fibrotic diseases from fibrosis associated with pneumonia to major, widespread fibrotic changes of the lung (George et al., 2020). A longitudinal study conducted by Wang Y. et al. (2020) on computed tomography (CT) manifestations during the course of COVID-19 pneumonia reported that, in the early stage of disease (first 11 days), ~85% of enrolled patients showed pulmonary ground-glass opacity and tissue consolidation. Later, between days 12 and 17, evolving from ground-glass opacity, a mixed pattern with lung architectural distortion appeared, characterized by perilobular abnormalities, suggesting the presence of secondary organizing pneumonia. Given that organizing pneumonia possibly evolves to fibrosis with progressive and permanent lung damage, this may indicate that some patients, although negative for COVID-19 at their discharge, can develop progressive, fibrotic irreversible interstitial lung disease (Figure 2A) (George et al., 2020; Wang Y. et al., 2020).

Furthermore, early analysis from patients with COVID-19 at hospital discharge suggests a high rate of lung fibrotic abnormalities (George et al., 2020). However, the relation between the presence of fibrotic lesions and prognosis is under debate. Some researchers indeed suggest that the presence of fibrosis indicates a resolution status of the disease and a good prognosis (Pan et al., 2020). Others instead suggest that fibrosis may progress resulting in pulmonary interstitial fibrosis disease indicating a poor prognosis (Kong and Agarwal, 2020). Similar fibrotic lesions were previously observed in patients surviving from respiratory diseases caused by coronaviruses (SARS-CoV and MERS-CoV) similar to the SARS-CoV-2 virus responsible of COVID-19. Long-term studies indicated that a third of SARS patients who recovered from severe infection were left with permanent lung damage (Cox, 2020).

Similarly, long-term studies will be required to establish the real prevalence of post-COVID-19 fibrosis. However, due to pandemic nature of COVID-19, some researchers underline the prompt need to identify early biomarkers in the disease course to identify patients who potentially progress to pulmonary fibrosis (George et al., 2020; Spagnolo et al., 2020), and suggest that the delivery of antifibrotic therapy, combined with anti-inflammatory treatment to mitigate the cytokine storm that seems to be responsible of severe ARDS thought to trigger the development of lung fibrosis, might have a role in preventing fibrosis progression after SARS-CoV-2 infection.

The development of anti-fibrotic drugs able to restore lung function in IPF patients remains an open challenge. Current antifibrotic therapy applied to patients with IPF, mainly represented by nintedanib and pirfenidone, can slow the progression of the disease and improve the rate of mortality, however they do not reverse the fibrotic process, and the only approach to effectively resolve pulmonary damage remains lung transplantation (Chase and Gallicchio, 2019). Therefore, there is a need for new therapeutics with anti-fibrotic effects on the injured lungs.

PDCs are widely considered as possible treatment for inflammatory-based fibrotic diseases. These cells, and their derivatives such as cell-derived exosomes and soluble factors, possess anti-inflammatory, anti-fibrotic, anti-apoptotic, and regenerative properties which altogether may synergistically contribute to stop and reverse fibrotic disease.

### PDCs and Derivatives in Animal Models of Pulmonary Fibrosis

The ability of PDCs and their derivatives to slow and/or reverse pulmonary fibrosis has been mostly investigated in mouse models of bleomycin-induced lung fibrosis that represents the most used to study pulmonary fibrosis and to test possible anti-fibrotic drugs (Peng et al., 2013). In this model, bleomycin induces oxidative stress specifically to alveolar epithelial cells leading to acute alveolar injury, and a consequent inflammatory response with immune cell infiltration in lung parenchyma and secretion of inflammatory and pro-fibrotic cytokines. Lung inflammation triggers the fibrotic response characterized by fibroblast activation to myofibroblasts, cells with high proliferation and responsible of excessive production of extracellular matrix that disrupts the normal lung architecture with loss of respiratory capacity (Peng et al., 2013). Given the involvement of an inflammatory trigger, it has been hypothesized that therapeutic approaches with a positive outcome in the bleomycin model might be beneficial also in COVID-19, both during the acute inflammatory phase and in preventing long-term fibrotic complications (George et al., 2020).

PDCs, including those from amniotic membrane, Wharton's jelly, and related derivatives, including cell secretome and secretome-derived exosomes, have been administered in animals with pulmonary fibrosis to evaluate their ability to prevent disease (delivery concomitant to fibrosis induction) (Cargnoni et al., 2009; Moodley et al., 2010; Murphy et al., 2011) and to evaluate their potential to cure the disease (delivery after fibrosis establishment) (Moodley et al., 2013; Vosdoganes et al., 2013).

#### Human Amniotic Epithelial Cells (hAEC)

hAEC and their derivatives are the most studied perinatal cells in experimental models of lung fibrosis. The antifibrotic effect of these cells have been investigated when delivered within 24 h from fibrosis induction by bleomycin (Moodley et al., 2010; Murphy et al., 2011) and when delivered at days 7 or 14 post bleomycin induction when lung injury was established (Moodley et al., 2013; Vosdoganes et al., 2013; Tan et al., 2018). However, globally these studies suggest that hAEC can not only slow down fibrosis progression, but also partly reverse the established fibrotic lesion. Furthermore, the lower levels of pro-inflammatory and pro-fibrotic cytokines (MCP-1, TNF-α, IL-1, IL-6, TGF-β), suggest that hAEC may exert their reparative effects by modulating the host inflammatory response.

Other studies demonstrate the ability of hAEC to interfere with specific immune cells involved in the bleomycin-induced inflammatory response. Murphy et al. (2012) observed that the therapeutic effect of hAEC depends upon the presence of functional macrophages and Tan and collaborators (Tan et al., 2015) demonstrated that hAEC treatment promotes the switch from M1 (pro-inflammatory activity), to M2 macrophages (associated with fibrosis resolution) characterized by lipoxin A4-dependent upregulated phagocytosis (Tan et al., 2017). The same group showed that hAEC can partly counteract T cell-mediated inflammation by increasing pulmonary levels of regulatory T cells (Treg) (Tan et al., 2015). Furthermore, they found that exosomes from hAEC, when delivered early after bleomycin-challenge, exerted an anti-inflammatory effect on macrophages and neutrophils, while a delayed delivery (7 days after bleomycin) was able to improve tissue-to-airspace ratio and reduce fibrosis (Tan et al., 2018), indicating that a cell-free treatment derived from hAEC (i.e., exosomes) can counteract both the pulmonary inflammatory phase and the following fibrotic phase induced by bleomycin challenge.

#### Human Amniotic Mesenchymal Cells (hAMSC)

In a model of pulmonary fibrosis induced by a dual bleomycin doses to better reflect the repeated injuries that underlie most lung diseases, Moodley et al. (2013) compared the antifibrotic potency of hAMSC, hAEC, and BM-MSC. All reduced infiltration of inflammatory cells in lung parenchyma and reduced lung levels of the profibrotic cytokine TGF-β. Interestingly, only hAMSC effectively reduced collagen deposition. In another study, hAMSC mixed with hAEC and human chorionic MSCs reduced the severity and extension of lung fibrosis and decreased neutrophil infiltration when delivered 15 min after bleomycin challenge (Cargnoni et al., 2009). Interestingly similar effects, together with improved blood gas parameters, were found when the hAMSC secretome was used (Cargnoni et al., 2012, 2014), indicating that hAMSC secrete active mediators crucial for tissue repair. More recently, hAMSC were found able to reduce collagen deposition, T cell lung infiltration, and inflammatory cytokine expressions also in rats with paraquat-induced lung fibrosis (He et al., 2020). Another recent study reported that administration of human placental MSC of fetal origin (hfPMSC), isolated from an unspecified region of placenta, in mice with bleomycin-induced lung fibrosis reduced collagen deposition and the production of pro-fibrotic cytokines by attenuating the dysregulation of MyD88/TGF-β signaling axis that is considered involved in the pathogenesis of pulmonary fibrosis in mice (Li et al., 2017). Finally, a very recent study demonstrates that hAMSC delivery in bleomycin-challenged mice reduces pulmonary B cell recruitment, retention and maturation, and counteract the formation and expansion of intrapulmonary lymphoid aggregates, thus dampening chronicization of lung inflammatory processes with a consequent reduction in fibrosis progression (Cargnoni et al., 2020).

#### Human Umbilical Cord Mesenchymal Stromal Cells (hUC-MSC)

Mesenchymal cells derived from the human umbilical cord (including Wharton's jelly, herein collectively referred to as hUC-MSC), have also been extensively investigated in experimental models of lung fibrosis. The first study performed with these cells reported that IV injection in immunodeficient mice (SCID) 24 h post-bleomycin challenge decreased lung inflammatory cell infiltrate and the expression of pro-inflammatory (MIF, TNF-α and INF-⋎) and pro-fibrotic cytokines (TGF-β). hUC-MSC also reduced collagen interstitial levels which could be correlated to the pro-degradative milieu created by increased MMP-2 levels and reduced their endogenous inhibitors (Moodley et al., 2009). Similar findings related to hUC-MSC ability to reduce collagen deposition were reported in a more recent study where hUC-MSC were administered twice (24 h and 7 days post-bleomycin) (Moroncini et al., 2018). In addition, hUC-MSC reduced the expression of IL-6 and levels of M2 macrophages.

hUC-MSC have been also used in the effort to revert pulmonary fibrosis induced by bleomycin in lung left lobe of rats. In this case, cells were administered at day 21 after bleomycin challenge. hUC-MSC transplantation reduced fibroblast activation and collagen accumulation associated with preserved alveolar structure and gas exchange (Chu et al., 2019). Furthermore, in contrast to Moroncini et al. (2018), hUC-MSC increased levels of pulmonary M2 macrophages producing matrix metallopeptidase 9 (MMP-9) involved in collagen degradation. Hyaluronan, released from hUC-MSC, appeared to be involved in macrophage polarization to an M2-like phenotype (Chu et al., 2019).

Recently, to improve the migration and homing of transplanted cells toward injured tissues, CXCR4 over-expressing hUC-MSC were IV injected in a murine model of radiation-induced lung injury (Zhang et al., 2019). A higher number of CXCR4-overexpressing hUC-MSC reached the injured lung tissues with respect to “normal” hUC-MSC and were more effective in decreasing the radiation-induced lung injury. Indeed, a marked reduction in the expression of the pro-fibrotic cytokine TGF-β1 and of α-SMA and collagen I was reported. Furthermore, CXCR4-overexpressing hUC-MSC preserved the expression of E-cadherin, suggesting that cell treatment can inhibit the radiation induced epithelial-mesenchymal transition (EMT) process and fibroblast activation into myofibroblasts. The same study reported that transplanted CXCR4-overexpressing hUC-MSC could express pro-Surfactant Protein (SP)-C, a marker of alveolar epithelial II cells, suggesting a possible differentiation of hUC-MSC into alveolar epithelium when transplanted *in vivo* one day after radiation (Zhang et al., 2019). Finally, MSCs derived from the umbilical cord vein (hUCV-MSC), not from the stromal tissue of umbilical cord, were transplanted in bleomycin challenged mice after previous exposure to oxygen peroxide (H_2_O_2_) and, similar to hUC-MSC, preserved alveolar spaces, reduced TGF-β and α-SMA expressions and myeloperoxidase activity, indicative of reduced lung inflammation (Mahmoudi et al., 2020).

## MSCs Anti-Microbial Effects

It is known that MSCs and other stromal cells present antimicrobial activities, as well as resistance or partial resistance to viral infection (Li et al., 2016; Khoury et al., 2020). Importantly, MSCs and other stromal cells including those of perinatal origin can exert antimicrobial effects either by limiting viruses/bacteria cell colonization and replication, or by limiting complications due to a dysregulated host response to infection such as sepsis. At the moment there are only a few studies reporting the antiviral and antimicrobial effects of perinatal stromal cells. Indeed, the most part of the effects herein reported were observed using other sources of MSCs, such as bone marrow, so we have highlighted those that resulted from studies conducted with perinatal MSCs. Thus, in the following sections we will provide an overview of the applications of MSCs for the treatment of viral infections and sepsis.

### Antiviral Properties of MSCs

Antiviral properties of MSCs have been studied in animal models of respiratory virus infections with controversial findings (Khoury et al., 2020). On one hand many studies report the ability of MSCs to counteract the damage caused by the virus exerting a protective action, putatively also through their immunomodulatory activity that is able to counteract an altered immune response. On the other hand, it should be emphasized that, other studies report how the susceptibility of MSCs to viral infection can alter their immunomodulatory properties while others report limited or negligible results upon MSCs treatment.

Indeed, in an immune-competent mouse model of H9N2 AIV-induced acute lung injury, (Li et al., 2016) reported how a single IV injection of BM-MSCs 30 min after the administration of the virus significantly improved physiological parameters with reduction in mortality, lung edema, and histologic injury. Furthermore, they reported reduced levels of inflammatory cytokines and chemokines (Li et al., 2016). On the other hand, systemic administration of BM-MSC in an immune-competent mouse model of lung injury by H1N1 infection, supplemented or not with the antiviral oseltamivir, did not improve overall survival or lung injury (Darwish et al., 2013). These findings where confirmed by another group that reported how BM-MSCs injection failed to prevent the damage induced by PR8 infection.

Furthermore, another study conducted on lung MSC isolated from 1 to 2 week-old specific pathogen free chickens lungs susceptible to influenza virus infection. The authors showed that MSCs infected with two avian influenza viruses, H1N1 and H9N5, became reservoirs and replication sites for the virus itself. Moreover, the lung MSCs changed their secretome profile with increased release of inflammatory cytokines such as IL-6 and IL-8 and became apoptotic (Khatri et al., 2010). Similar findings were obtained by Cheung et al. (2016) who reported how the respiratory syncytial virus (RSV) was able to infect both human BM-MSCs as well as UC-MSCs modifying their immunomodulatory properties. Indeed RSV-infected MSCs had increased expression of IL-1β, IL-6, IL-8, and SDF-1, and also showed an altered capacity to affect PBMC proliferation (Cheung et al., 2016).

However, other groups have reported that MSCs prevented influenza-induced thrombocytosis and partially reduced the viral load (Gotts et al., 2014). The protective effect exerted by the MSCs on preserving vascular endothelial integrity was also reported in *in vivo* model of lung hemorrhagic shock (Pati et al., 2011).

In addition, several studies reported the efficacy of MSC treatment in improving the survival of animals with viral infection. Chan et al. (2016) reported how MSC injection strongly improved the dysregulated alveolar fluid clearance induced by the avian virus H5N1. Furthermore, the authors reported how the treatment with MSCs improved alveolar fluid clearance by upregulating the expression of the sodium chloride channel that is usually downregulated by the viral infection (Chan et al., 2016). Importantly, substantial differences in the beneficial effect exerted by MSCs according to the age of the animal were observed. Indeed, MSC injection induced a general improvement in old mice (8–12 months old) by reducing the mortality, lung edema, the total area of lung lesion. In parallel, researchers observed a reduction in the amount of total infiltrating lymphocytes. These effects were not appreciable in young mice (6–8 weeks old) (Chan et al., 2016). This age dependent sensitivity has also been observed in COVID-19 patients. In fact, the severity of the disease increases in aged people while children often develop mild symptoms (Liu K. et al., 2020). However, it still remains to be understood whether this effect is related to reduced levels of Angiotensin-converting enzyme 2 (ACE2) receptor expressed by children, to the immune-senescence appreciable in elderly people, or to other phenomena (Liu K. et al., 2020; Nikolich-Zugich et al., 2020). Conversely, Li et al. (2016) demonstrated how MSC treatment was effective in reducing the lung injury in young (8 weeks old) immunocompetent mouse model of viral H9N2-AIV infection, in parallel reducing the amount of inflammatory cytokines present at serum level.

In a comparison paper, hUC-MSCs were more effective than BM-MSCs in improving the overall survival of H5N1-infected mice. hUC-MSCs were shown to release angiogenin-1 and hepatocyte growth factors that were able to enhance the alveolar fluid clearance, thus reducing lung edema and inflammation (Loy et al., 2019). However, despite these results, MSC treatment was not found to be associated with a general reduction of the viral load (Khoury et al., 2020). The ability of MSCs to act positively in counteracting the damage caused by viral infection was not limited to the cell therapy *per se*, but as aforementioned positive results were obtained also by the use of the cell secretome (Krasnodembskaya et al., 2010) as well as with the extracellular vesicles (EVs) (Börger et al., 2020). Indeed, MSC-EVs were able not only to suppress the viral replication in lung epithelial cells (Khatri et al., 2018), but also to exert anti-inflammatory effects (Willis et al., 2018; Park et al., 2019; Varkouhi et al., 2019). Conversely, in an *in vitro* study, the immunomodulatory effect of hUC-MSCs was reported to be responsible for the reduced cytotoxic activity of a subset of T lymphocytes, Vγ9Vδ2 T lymphocytes, against H1N1-infected A549 cells (Liu et al., 2015). On the other hand, it has also been reported that MSCs did not affect the viral-specific T lymphocyte activity (Karlsson et al., 2008). Indeed, Karlsson et al. (2008) found that MSCs co-cultured with virus specific EBV or CMV cytotoxic T lymphocytes maintained their cytotoxic activity. These results were also confirmed by a clinical report highlighting how patients who received MSCs for graft vs. host disease (GVDH) treatment still maintained the capacity to responded to CMV viral infection (Karlsson et al., 2008).

### Anti-septic Properties of MSCs

For many aspects COVID-19 pathogenesis is comparable to sepsis. Indeed, sepsis is defined as a life-threatening organ dysfunction caused by a overreacted host response to infection (Singer et al., 2016). As a matter of fact, COVID-19 patients are often hospitalized for severe pneumonia that rapidly deteriorates to multi organ failure as a consequence of infection spread and cytokine storm (Jose and Manuel, 2020).

The pathogenesis of both sepsis and COVID-19 are indeed characterized by the massive infiltration of immune cells to the target organ or tissue, increased levels of cytokines and signaling molecules released which attract more immune cells that triggers a feedback loop that exacerbates inflammatory response causing the formation of tissue damage and potentially leading to multi-organ failure and death (Singer et al., 2016; Jose and Manuel, 2020). In line with this, one study demonstrated that SARS-CoV2 infection was reported to trigger acute kidney failure in 20% of cases analyzed (Arentz et al., 2020). Another case report highlighted that over 30% patients with COVID-19 developed liver injury (Bhatraju et al., 2020). In both studies the authors report that patients undergo septic shock.

MSCs have been reported to improve survival in different preclinical models of sepsis (Lombardo et al., 2015). They have also been shown to reduce the deleterious effects determined by the cytokine storm typical in sepsis model. These findings were confirmed by the reduction of plasma inflammatory cytokines after administration of BM-MSCs in different mouse models of sepsis, such as intraperitoneal injection of LPS (Xu et al., 2007; Saeedi et al., 2019) or the caecal ligation and puncture (Mei et al., 2010; Luo et al., 2014). A study using hAMSCs also reported that treatment of mice with cecum-puncture-induced sepsis protected mice against death caused by diffuse peritonitis (Parolini et al., 2014).

These effects were also confirmed in a mouse model of ARDS and sepsis due to *Escherichia coli (E. coli)*-induced pneumonia sepsis (Horie et al., 2020). Treatment with a subpopulation of hUC-MSCs, more specifically CD362+hUC-MSCs, resulted in the reduction of bacterial infiltration and injury, thus ameliorating inflammatory marker levels (Horie et al., 2020). Similar findings were also obtained in rat models of ARDS or ARDS combined with severe sepsis (ARDS-SS) syndrome obtained upon caecal-ligation and puncture. In both models, hUC-MSC administration reduced the serum levels of inflammatory cytokines, and slightly reduced the percentages of inflammatory cells in ascites and kidney parenchyma thus improving the overall survival (Lee et al., 2017). Another study reported that the beneficial effect exerted by hUC-MSC transplantation was due to the release of the anti-microbial peptide LL-37 (Zhu, 2017).

The anti-microbial effect of MSCs is not limited to the MSC *per se*. Indeed, beneficial results have also been obtained after injection of the secretome derived from BM-MSC culture. These effects were strictly related to the increased expression of the antimicrobial peptide LL-37 by MSCs primed with *E. coli* (Krasnodembskaya et al., 2010). Other bioactive molecules released by MSCs are lipocalin-2, that has been shown to regulate the activation of neutrophils and thus improve bacteria clearance (Gupta et al., 2012), and beta-defensin-2. Importantly, the injection of hUC-MSCs in a mouse model of *E. coli*-induced acute lung injury improved the bacteria clearance by the release of beta-defensin-2 upon the direct triggering of the TLR4 (Sung et al., 2016).

The beneficial effects obtained after MSC injection to treat sepsis have been shown to be related to their strong immunomodulatory properties. Indeed, MSCs were able to reduce the plasma levels of different inflammatory cytokines thus improving the lung histology (Gonzalez-Rey et al., 2009; Shin et al., 2013; Sepúlveda et al., 2014), as well as cardiac tissue quality (Weil et al., 2010; Manukyan et al., 2011), in different *in vivo* models of sepsis. Importantly, MSC treatment can reduce the infiltration of inflammatory cells like neutrophils, macrophages, and T lymphocytes to the target organs in animal model of sepsis (Mei et al., 2010; Anderson et al., 2013; Hall et al., 2013; Zhao et al., 2014). Moreover, reduced IL-6 and IL-1b, two relevant inflammatory cytokines in sepsis that it have been reported to play a relevant role in COVID-19 [as highlighted by the different clinical trials using different monoclonal antibodies targeting IL-6 (Tocilizumab) (De Luna et al., 2020; Toniati et al., 2020) as well as IL-1b (Anakinra) (Huet et al., 2020)], was also observed (Wilson et al., 2015; McIntyre et al., 2018). Also, IL-10 plays a relevant role in modulating the immune response. Indeed, COVID-19 patients often present high plasma concentration of this cytokine (Chiappelli et al., 2020; Huang et al., 2020), and MSCs have been shown to modulate the serum levels of IL-10 by stimulating its production by immune system cells. Indeed, it has been reported that IL-10 is not directly produced by MSCs but is a consequence of the macrophage skewing toward anti-inflammatory M2 macrophages; an effect that seems to be triggered by the prostaglandin E2 (PGE2) released by MSCs (Nemeth et al., 2009). Furthermore, this effect was also confirmed in *in vivo* studies. Indeed, the intraperitoneal injection of M2 macrophages, induced by the *in vitro* co-culture with BM-MSCs, in septic mice were able to improve the overall survival (Anderson et al., 2013). The effects were due to a general systemic reduction of immune cell infiltration in different organs (Anderson et al., 2013). Noteworthy, when septic mice depleted for monocytes or neutrophils were treated with MSCs, no beneficial effects were reported, thus highlighting the crucial role of the interactions of MSCs on immune cells in order to exert a therapeutic effect (Nemeth et al., 2009; Hall et al., 2013).

Overall, there are numerous experimental evidences that support the application of PDCs in the treatment of COVID-19. The intrinsic properties of PDCs, notorius for their immunomodulatory properties, seem to perfectly mitigate the etiopathological characteristics of COVID-19 (Figure 2B).

## Author Contributions

AP, AC, MS, and AS: manuscript writing. AS and GK: manuscript writing and editing. RO and OP: conception and design, financial support. All authors contributed to the article and approved the submitted version.

## Conflict of Interest

MS, GK, and RO are employed by the company Pluristem Ltd. The remaining authors declare that the research was conducted in the absence of any commercial or financial relationships that could be construed as a potential conflict of interest.
